# Melanoma of the Urinary Bladder: A Review of the Literature

**DOI:** 10.1155/2014/605802

**Published:** 2014-01-12

**Authors:** Anthony Kodzo-Grey Venyo

**Affiliations:** North Manchester General Hospital, Delaunays Road, Manchester, UK

## Abstract

*Background*. Melanomas of the urinary bladder and urethra are rare. * Aims*. To review the literature on the disease. * Methods*. Various Internet databases were used to identify reported cases of the disease. *Results*. Less than 30 cases of primary melanoma of the urinary bladder and urethra have been reported in the literature and they have been associated with melanosis and commonly with metastases. The lesions may be primary or metastatic with no gender preference. The diagnostic features include pigmented raised lesions which histologically exhibit spindled or epitheliod cells, necrosis, mitotic figures, and atypical melanocytes. Immunohistochemically they stain positively with S100; HMB45; and other melanocyte markers, but negatively with Keratin and Vimentin. The treatment involves excision and possibly IL-2. The prognostic factors include size and depth of invasion as well as metastatic lesions. * Conclusions*. Less than 30 cases (about 24 cases) of the disease have been reported. There are also reports of metastatic melanomas of the urinary bladder emanating from primary melanomas originating elsewhere. Diagnosis of the primary disease is based upon the histological appearance of the lesion, positive staining with S100 and HMB45, and evidence of absence of melanoma elsewhere. Primary melanoma of the bladder is usually a fatal lesion.

## 1. Introduction

Primary malignant melanoma of the genitourinary tract is a rare lesion, which most medical practitioners including urologists would never encounter in their working lives and its diagnosis may be difficult for most practitioners. The ensuing document has discussed various aspects of primary melanoma of the urinary bladder including the differential diagnoses.

## 2. Methods

Various internet databases including Pub Med, Medline, Google Scholar, Google, and Educus were used to identify reported cases of melanoma of the urinary bladder in order to document the presentation, investigation, management, and treatment outcome of the disease. In all, 64 references were identified which were used to write the literature review.

## 3. Literature Review

### 3.1. Overview

*Definition*. Primary melanoma tumours of the urinary bladder are rare and less than 50 cases have so far been reported in the literature [1]. Melanomas of the urinary bladder which are associated with melanosis and metastases are much common in melanoma of the urinary bladder [1]. Urinary bladder at times may be affected or involved by secondary/metastatic melanoma.

*Epidemiology*. Melanoma occurs in patients who are aged between 44 years and 81 years old [1]. Melanoma of the urinary bladder occurs in both male and female and there is no gender preference [1]. 

*Sites*. Melanoma may be found in the urinary bladder and urethra [1]. 

*Aetiology*. Melanoma of the urinary bladder may be either a metastatic lesion or a primary lesion. 

*Clinical Features*. The most frequent presenting symptom is visible haematuria. [1] Almost all the reported cases of melanoma of the urinary bladder have been associated with death of the patient [1].

*Prognostic Factors*. The prognosis of melanoma of the urinary bladder depends upon the following: presence of metastatic lesions; and size and depth of invasion of the melanomas.

*Treatment*. Melanoma of the urinary bladder can be treated by means of excision and possibly by means of IL-2 [2].

*Clinical Features*. Cystoscopy examination of the urinary bladder would reveal pigmented raised lesions [1]. The differential diagnosis of melanoma of the urinary bladder include: endometriosis, melanoma, and sarcoma.

*Macroscopic Features*. Melanoma of urinary bladder occurs throughout the bladder with variable pigmentation [3].

*Microscopic Description*. Microscopic examination of malignant melanoma of the urinary bladder tends to reveal the following features.

- Spindled or epitheliod cells. The epitheliod cells often have marked pleomorphism, abundant cytoplasm, and “prominent,” eosinophilic nucleoli [1].
- Necrosis and mitotic figures are common [1].
- Often atypical melanocytes can be seen [1].

*Cytology Features*. Cytology examination in cases of malignant melanoma of the urinary bladder reveal the following features.

- Cells with pleomorphic nuclei singly or in clusters may be seen [1].
- Spindle cells can be seen [1].
- Often cytoplasmic melanin pigment and prominent eosinophilic nucleoli can be seen [1].
- Malignant cells may be seen and some of the cells would be seen to contain cytoplasmic melanin; also macrophages containing melanin may be seen [1, 4].

*Positive Immunohistochemical Stains*. Melanoma of the urinary bladder stains positively for the following stains:

- S100 [1],
- HMB45 [1],
- other melanocyte markers [1].

*Negative Immunohistochemical Stains*. Melanoma of the urinary bladder stains negatively for the following tumour markers:

- keratin [1],
- vimentin [1].

*Differential Diagnosis*. The differential diagnoses of primary melanoma of the urinary bladder include

- sarcoma which is a poorly differentiated carcinoma [1].

*Metastatic Melanoma*. In cases of metastatic melanoma a clinical history of primary melanoma may exist, although some primaries spontaneously regress so this may be difficult to conclusively establish and no bladder melanosis can be seen [1].

### 3.2. General Observation and Narrations from Reported Cases of Melanoma of the Urinary Bladder

#### 3.2.1. General Observation

To the knowledge of the author less than 30 cases of melanoma of the urinary bladder have so far been reported in the literature and these have been treated by means of various treatment approaches. The various treatment options that have been reported for cases of primary melanoma of the urinary as illustrated in Table 1 include transurethral resection of bladder tumour, partial cystectomy, radical cystectomy, chemotherapy, local excision and radiotherapy and chemotherapy, cystectomy and radiotherapy, interferon *α*, and intravesical instillation of Bacillus Calmette-Guerin and there is no consensus of opinion regarding the best management option.

#### 3.2.2. Narrations from Reported Cases of Melanoma of the Urinary Bladder

Kerley et al. [3] reported an 80-year-old woman who developed vulvar melanosis and malignant melanoma of the labia majora and clitoris and she underwent simple vulvectomy. She subsequently developed melanosis of the urinary bladder and she presented 3 years later with multifocal malignant melanoma which involved the vagina, urethra, and urinary bladder in a background of extensive melanosis with variable degrees of atypia. She underwent radical surgery but she died 18 months later with liver metastases and liver failure. Kerley et al. [3] stated the following.

- Malignant melanoma is uncommon in the vulva and vagina and it is rare in the urinary bladder.
- Their case illustrated the previously described association between melanosis and malignant melanoma.
- The unusual features were the widespread distribution of the melanosis and malignant melanoma (vagina and bladder) and the subsequent development of multifocal malignant melanoma.

Pacella et al. [5] in 2006 stated that at the time of the report of their case of primary malignant melanoma of the urinary bladder 18 cases of primary malignant melanoma of the urinary bladder had been reported in the literature. Pacella et al. [5] reported an 82-year-old man who underwent trans-urethral resection of a bleeding tumour on the posterior wall. The histological diagnosis of the resected lesion was malignant melanoma of the urinary bladder. Pacella et al. [5] stated that there was no history of previous or regressed cutaneous malignant melanoma. The margins of the urinary bladder lesion contained atypical melanocytes similar to those commonly seen in the periphery of primary mucous membrane lesions. Clinical studies and radiological examinations were negative for other primary sites of melanoma. The patient had a bladder recurrence which was consistent with primary tumour and he died of widespread disease 9 months after the diagnosis. Pacella et al. [5] detailed out their report as follows.

An 82-year-old Caucasian man was admitted because of gross haematuria. The patient had a history of a single episode of superficial urothelial bladder cancer that occurred 3 years previously and that was treated with transurethral resection alone. Subsequent followup consisted of cystoscopy and urinary cytology and these were normal till the last control, which had been held 2 months before his haematuria recurred. The patient's medical and surgical history included acute myocardial infarction, carotid artery disease, and supra-pubic prostatectomy. His general physical examination was unremarkable. His routine blood tests were normal. He had intravenous pyelography which demonstrated a filling defect of the bladder. He underwent cystoscopy which revealed a darkly stained tumour of the posterior bladder wall that resembled a voluminous organized coagulum. A transurethral resection of the lesion was performed reaching deep muscular layers of the bladder, and a sample of peritumoural mucosa was obtained. Pathologic examination of the resected lesion revealed a tumour composed of epithelial atypical, pigmented melanocytes interspersed with transitional epithelium. These cells which were found to have invaded lamina propria and muscular layers were positive under S-100 and HMB-45 immunostaining. Atypical melanocytes were seen in urothelial mucosa at the tumour margins. Exhaustive investigation was undertaken to exclude other primary sites of melanoma. Dermatologic, ophthalmologic, otorhinolaryngologic, and proctologic evaluations were negative. Upper gastrointestinal endoscopy and colonoscopy were negative. Abdomen, chest, and brain computed tomography (CT) scans were negative. A bone scan was negative. Diagnosis of primary malignant melanoma of the urinary bladder was made. Pacella et al. [5] reviewed published scientific literature and discussed the clinical case with their oncologist who suggested no adjuvant therapy because of the patient's age, comorbidity, and poor prognosis despite treatment. They informed the patient about the therapeutic options, such as radical cystoprostatectomy plus immunochemotherapy, immunochemotherapy alone, observation, and treatment of symptoms. The patient did not accept any further treatment. Three months later, he developed further haematuria and he underwent cystoscopy which showed bleeding nearby the site that was previously resected without clear evidence of a local recurrence. A biopsy and an extensive coagulation of the area were then performed; histological examination diagnosed a recurrence that was consistent with primary tumour. A CT scan was then performed and this showed two nodular lesions of the liver, a nodule in each adrenal gland, two nodules of the right lung, and multiple mediastinal enlarged lymph nodes. A subtle and continuous haematuria persisted and the patient died 9 months after diagnosis as a result of complications caused by widespread metastases. Autopsy was not performed.

El-Ammari et al. [6] in 2011 reported a 71-year-old Moroccan male who presented with a 2-month history of gross haematuria. There was no past history of calculous disease or flank pain. He had been smoking 50 cigarettes a day for the preceding 40 years prior to his presentation. There was no history of contact with aniline dyes or other chemicals. His physical and paraclinical examinations were normal. He had ultrasound scan which revealed a 5 cm size heterogeneous mass which was located in the trigone of the urinary bladder. He underwent cystoscopy which showed a 50 × 40 × 10 mm darkly pigmented flat papillary lesion of the bladder trigone. He underwent deep transurethral resection of the bladder lesion. Histological examination of the resected specimen showed pigmented malignant melanoma. Exhaustive investigation was undertaken to exclude other primary sites as the source of the melanoma. Careful dermatological examination including Wood's light examinations of the “entire body” and ophthalmologic evaluations were negative. Upper gastrointestinal and barium enema studies were negative. He had computed tomography scan of the abdomen, including liver and spleen, and this was normal, as was CT scan of the brain and chest. He also had bone scan which was negative. The diagnosis of primary malignant melanoma of the urinary bladder was made. The patient died five months later before any radical treatment could be performed. El-Ammari et al. [6] stated that to their knowledge at the time of their publication in 2011 19 cases of primary melanoma of the urinary bladder had been reported worldwide in the literature.

Ainsworth et al. [7] stated the following.

- Malignant melanoma of the genitourinary tract is most commonly secondary to melanoma which had occurred in other sites.
- A number of cases of primary melanoma of the urinary bladder had previously been reported and, in such cases, a detailed patient history, careful examination of the patient's skin, and evaluation for other visceral primary sites are necessary to confirm the primary nature of the tumour [7].

Khalbuss et al. [4] stated that the histogenesis of primary bladder melanoma is uncertain and that an origin from cells of the neural crest had been proposed [4]. Ainsworth and associates in 1976 were the first observers to carefully delineate the criteria for defining primary malignant melanoma of the urinary bladder. These include

1. careful physical examination including the skin with Wood's light together with detailed history to exclude cutaneous melanoma,
2. exclusion of visceral melanoma following exhaustive evaluation,
3. pattern of recurrence consistent with primary melanoma of the urinary bladder,
4. histologically proven primary atypical melanocytes [2].

Wheelock [8] was the first to report a primary melanoma of the bladder in 1942; then Su and Prince [9] reported one case in 1962, and this was followed by Anichkov and Nikonov [10] who reported two cases of primary bladder melanoma but in neither case were exhaustive studies undertaken to exclude other sites' of primary melanoma [9, 11, 12]. Primary bladder melanoma is extremely rare, and perhaps none of those previously reported cases truly arose from the bladder. Several treatments were proposed to deal with such a rare tumour. Transurethral resection of the lesion, partial cystectomy, radical cystectomy, chemotherapy, and radiation therapy had been used to treat melanoma of the urinary bladder [5, 7, 9, 12]. It was stated by some authors [5, 7, 9, 12] that, in all patients with localised tumour, radical surgery seemed to be the therapy of choice, although to date none of the patients survived more than three years despite cystectomy characterizing the poor prognosis of the tumour [5, 7, 9, 12]. In view of the fact that the tumours recur after local excision in many cases, adjuvant chemotherapy associated with radiotherapy may improve patients' survival [13]. Interferon is another alternative for the management of melanoma and showed 10% remission rate in metastatic cutaneous melanomas [14]. Despite this variety of treatments, the prognosis is bad. Ainsworth's patient was treated with radical cystectomy followed by chemotherapy and radiotherapy and developed local recurrence after 14 months [7]. In 2006, Pacella et al. [5] reported an 82-year-old patient who presented a local recurrence and died nine months after the diagnosis. The patient reported by El Ammari et al. [6] died of widespread disease two months after diagnosis. However, Neiderberger reported 18 months disease-free survival in a primary bladder melanoma patient, who was treated with radical cystectomy alone [12]. In all reported cases, the tumour was deeply invading the muscle which may explain the very bad prognosis [5, 7, 9].

DeKernion et al. [15] reported an isolated metastasis of malignant melanoma to the urinary bladder of a patient which was successfully eradicated by transurethral intralesional injection of Bacillus Calmette Guerin (BCG). DeKernion et al. [15] stated the following.

- Total destruction of the tumour was confirmed by subsequent excision.
- Lymphocyte blastogenesis studies revealed no significant alteration in immunocompetence secondary to the therapy, except for an increased responsiveness to PPD.
- There was no evidence of presence of blocking factors following therapy; cytotoxicity against MLA-14 melanoma cells sharply increased after the intralesional injection.
- Humoral antimelanoma antibody levels, determined by complement fixation, were decreased before the intralesional therapy but increased markedly immediately following the transurethral BCG injection.

Lee et al. [2] stated the following.

- Recombinant interleukin-2 (IL-2) had demonstrated antitumor activity and durable clinical responses in patients with metastatic melanoma.
- Careful screening and selection of appropriate patients had improved the safety profile of IL-2 administration.
- Gross haematuria would ordinarily preclude the safe delivery of IL-2.

Lee et al. [2] reported a case of metastatic melanoma to the bladder which presented with haematuria. A complete resection was performed and subsequently allowed the administration of high-dose, bolus IL-2. The combination of resection and IL-2 therapy resulted in a partial response which was maintained for more than 18 months. Lee et al. [2] suggested that symptomatic bladder melanoma should be aggressively treated to allow for systemic immunotherapy, which can provide durable responses.

Vasdev et al. [16] reported a 58-year-old, previously healthy male who presented in February 2004 with an eight-week history of a painless soft tissue mass lesion on the right buttock, going into the right side of the perineum. There were no associated symptoms.

He had clinical examination which confirmed a nontender 5 cm × 5 cm soft mobile mass, with no regional lymph nodes. His investigations were normal apart from a pelvic MRI, which surprisingly showed a large soft tissue mass lesion on the left side of the urinary bladder. He had cystoscopy which showed a solid mass lesion with normal overlying mucosa. He subsequently underwent trans-urethral resection of the bladder tumour (TURBT) and excisional biopsy of the buttock lesion. Histological examination of the specimens confirmed malignant melanoma, with metastasis to the urinary bladder. He then underwent partial cystectomy and excision of an enlarged mesenteric lymph node intra-operatively, which turned out to be involved. Four courses of systemic chemotherapy in the form of Tropisetron 5 mg, dexamethasone 8 mg, doxorubicin 150 mg, and Dacarbazine 1700 mg were started. He had been under regular followup with no evidence of tumour metastasis.

Some authors [17, 18] stated that most of the secondary malignant melanomas of the urinary bladder are asymptomatic. However, some may present with irritative lower urinary tract symptoms and/or haematuria.

A number of authors [19–26] stated that malignant melanoma is known to metastasize to different organs, including stomach and oesophagus and uncommonly to the urinary bladder as one of the rare sites of secondaries.

Menéndez López et al. [27] stated that, with regard to the time of presentation, secondaries of malignant melanoma could present simultaneously or after a period of time, which could be years prior to its clinical presentation.

Vasdev et al. [16] stated that the treatment of this rare condition is still controversial. Nevertheless, the aim of treatment is symptomatic relief, if this is the case. This is in the form of endoscopic management and/or systemic chemotherapy. In addition, reports have been made on intra-lesional injection of BCG treatment of the disease, with good early results. However, the long-term results are not available [15].

Menéndez López et al. [27] reported the case of a 75-year-old male with a history of malignant melanoma in the scapular region which was excised 7 years before he presents with lower urinary tract symptoms (LUTS), haematuria, and a hypogastric mass, 12 cm in diameter, which was located superficially and anterior to the bladder. He underwent partial cystectomy for excision of the mass, prostate adenomectomy, and lithiasis extraction. Pathological study revealed neoplastic cell proliferation with tendency to form sparse round nests or sheaths. The tumour cells had wide, polygonal cytoplasms and occasionally melanocytic pigment. Protein S-100 detection by immunohistochemical tests was positive. The final diagnosis was metastatic malignant melanoma. Menéndez López et al. [27] concluded the following.

- Bladder metastatic disease is unusual and rarely clinically evident. Nevertheless, it was frequent for bladder metastases to be caused by melanoma.
- Bladder metastatic lesions are rarely symptomatic, although approximately 15% of the cases are symptomatic, and haematuria is the most common presentation.
- Although radical cystectomy seems to be the treatment of choice in potentially curable patients with solitary metastasis, to date no patient has survived more than three years, demonstrating the aggressive natural history and ominous prognosis of this disease.

Despite the rarity of primary malignant melanoma of the urinary bladder, genitourinary system metastases of the bladder melanoma have a relatively higher incidence. Renal (45%) and vesical (18%) metastases had been found in patients deceased because of melanoma [5]. Therefore, discrimination between primary and metastatic melanomas of the bladder is crucial. Ainsworth et al. [7] and Siroy and MacLennan [28] established some diagnostic criteria for primary bladder tumours:

1. absence of any previous skin lesion, or
2. cutaneous malignant melanoma, or
3. primary visceral malignant melanoma,
4. recurrence pattern showing consistency with the primary tumour diagnosis,
5. atypical melanocytes at the tumour margin on microscopic examination.

In the literature including the most recent review of the literature by Siroy and MacLennan [28] the number of cases with primary melanoma of the bladder amounted to 19 at the time of the literature review [28]. However, all the cases reported in the literature as primary melanoma of the bladder do not appear to fit in these criteria of primary vesical melanoma. Its initial clinical presentation is haematuria, as seen in other types of bladder carcinomas. Nevertheless, haematuria is a clinical sign of locally advanced disease [29]. Some patients present with lower urinary tract symptoms. During advanced stages of the disease, clinical symptoms peculiar to metastatic disease can be observed.

Cystoscopy is the primary diagnostic modality. Cystoscopy usually reveals a dark pigmented mass with varying dimensions. Mucosal layer surrounding the tumour mass has a dark brown appearance, while the mucosa distant from the lesion has a pinkish white colour [7]. Diagnosis is made with histopathological examination of the biopsy material. Immunohistochemical studies shorten and facilitate diagnostic work-up.

Despite treatment alternatives including transurethral resection, partial and radical cystectomy, radiotherapy, immunotherapy, and chemotherapy, overall it has a poor prognosis. Transurethral resection is curative for lesions restricted to the epithelium, and actually the definitive cure could be achieved by radical cystectomy [5, 28]. When surgery is contraindicated or chemotherapy is not tolerated because of its side effects, radiation therapy and immunotherapy with interferon alpha can be applied [28]. Despite all these treatment alternatives, the prognosis is poor and the patients are generally lost within 3 years because of metastatic complications.

Sathiyamoorthy and Ali [30] stated the following.

- Malignant melanoma has an unusual predilection for metastasis to the small bowel, sometimes several years after the original diagnosis.
- In patients who have had a cystoprostatectomy followed by an ileal conduit, metastatic melanoma to the ileal conduit can present in urine cytology.

They presented a rare case of metastatic malignant melanoma in neobladder urine in a patient who had undergone a cystoprostatectomy for high grade urothelial carcinoma of the bladder and prostatic adenocarcinoma of Gleason grade 3 + 3 and two excisional procedures for cutaneous malignant melanoma. He presented with persistent haematuria and urinary tract infections unresponsive to treatment. His urine cytology revealed single large atypical cells, with large nuclei, prominent nucleoli, and cytoplasmic melanin pigment. Subsequent surgical resection revealed two areas of metastatic melanoma in the ileum, one of them being in the ileal conduit. The tumour cells were immunoreactive for S-100 protein, Melan-A, and HMB-45 and were negative for CAM5.2 and cytokeratins 7 and 20.

Charfi et al. [31] stated that plasmacytoid variant of melanoma is reported in only rare cases. They reported a 54-years-old man who was admitted because of enlarged lymph nodes in the lumbar region. A provisional diagnosis of plasmablastic lymphoma/plasma cell myeloma was considered. He was found to have a urinary bladder polyp which was removed. Microscopic examination of the polyp demonstrated dense plasmacytoid cells infiltration with pigment deposits. Immunohistochemical study of the polyp showed strong expression of HMB45, Melan A, and vimentin. There was focal positivity with S100 protein and CD138/syndecan-1. The diagnosis of metastatic plasmacytoid melanoma was finally established. Charfi et al. [31] further stated the following.

- Clinical examination revealed an esophageal melanoma with melanosis which supported its primary location.
- Although rarely, melanoma especially plasmacytoid variant may express plasma cell markers which may lead to erroneous diagnosis of plasma cell proliferation.
- Careful morphological examination for melanin pigment and the use of panel of melanocytic markers are helpful for diagnosis.
- Plasmacytoid variant of melanoma is a rare finding [32–37] which may mimic many other entities especially plasma cell proliferation.
- The use of immunohistochemistry in the diagnosis of such tumours is primordial.

Some authors [7, 38] stated that primary malignant melanoma of the urinary bladder is known to occur very rarely in the urinary tract, especially in the bladder. However, metastases of malignant melanoma to the genitourinary tract are relatively common. Approximately 45% of patients dying of melanoma have kidney metastases and 18% have bladder metastases [38]. Stein and Kendall [39] stated that primary malignant melanoma of the genitourinary tract is rare and this accounts for less than 1 percent of all malignant melanomas. Ainsworth et al. [7] stated that, while, primary urethral melanoma had been well described, reports of primary urinary bladder melanoma had generated some confusion. Therefore, it is very important to distinguish primary from secondary melanomas of the bladder. Ainsworth et al. [7] as well as Stein and Kendall [39] postulated the ensuing criteria for considering a melanoma of the urinary bladder a primary lesion:

- no history of previous cutaneous lesion,
- no evidence of regressed cutaneous malignant melanoma,
- no evidence of other visceral primary melanoma,
- pattern of recurrence should be consistent with the region of initial malignant melanoma,
- margins of bladder lesion should contain atypical melanocytes similar to those seen in the periphery of primary mucous membrane lesions.

Wheelock [8] in 1942 reported a 67-year-old white woman who had a vaguely described lesion at the “bladder neck and urethra.” However, Niederberger and Lome [12] were of the opinion that the case which was reported by Wheelock [8] represented the more common primary urethral malignant melanoma with extension into the urinary bladder. Niederberger and Lome [12] stated that, in the case which was reported by Su and Prince in 1962 [9], a scalp lesion was found three weeks pursuant to cystectomy. They, therefore, formed the opinion that this metastatic pattern indicated the likely secondary nature of the urinary bladder melanoma.

Niederberger and Lome [12] reviewing details of some other cases which were reported as “primary melanomas of the urinary bladder” and these in their opinion were most likely secondary or metastatic melanomas. They stated that Willis et al. [11] used history, clinical examination, and visceral studies to exclude secondary melanoma. Nevertheless, recurrent tumour was found on cranial computed tomography scan, and this computed tomography scan finding in their opinion represented a pattern of metastasis and the pattern was compatible with secondary melanoma.

Niederberger and Lome [12], furthermore, stated that Anichkov and Nikonov [10] reported 2 cases in 1982, but in their opinion in neither case were exhaustive studies carried out to exclude other sources of primary melanoma. Additionally Niederberger and Lome [12] felt that possibly out of the six cases of primary melanoma of the urinary bladder that were reported prior to their reported case of primary melanoma of the urinary bladder perhaps only 1 case represented genuine primary melanoma of the urinary bladder.

Pacella et al. [5] were also of the opinion that the diagnostic criteria are strict but not all cases reported in the published literature are definitely primary melanomas of the bladder. They were of the view the following.

- The case reported by Su and Prince was likely not a primary because a scalp melanoma was found after cystectomy [9] in 1982.
- Anichkov and Nikonov [10] did not perform a complete exhaustive clinical study and the diagnosis of primary bladder melanoma was, therefore, questionable.
- The only case of a long survivor was reported in 2000 by Garcia Montes [38]. A malignant melanoma of the bladder was diagnosed by transurethral resection. The atypical melanocytes did not infiltrate the lamina propria or the muscular wall. There was no evidence of atypical melanocytes in the urothelium mucosa at the tumour margins, therefore excluding the diagnosis of primary melanoma according to Ainsworth's criteria. Furthermore no other sites of melanoma were found after a meticulous clinical study and the patient was disease-free after 144 months and it is reasonable to suppose that patient was cured by surgery.

Pacella et al. [5] stated the following.

- Despite the availability of several therapeutic options, including trans-urethral resection, partial and total cystectomy, radiotherapy, chemotherapy and immunotherapy with interferon-alfa, the prognosis, excluding the latter report, is still poor (see Table 1 for a list of some of the reported cases of primary melanoma of the urinary bladder, their treatment and outcome).
- The majority of patients with primary melanoma of the urinary bladder presented with haematuria, which must be regarded as a late symptom of a locally advanced melanoma, and, therefore, a bleak prognosis could be explained by a subsequent delayed diagnosis. If such tumours could be treated before they became deeply infiltrative, prognosis might be improved by radical treatment. In the case of a lesion confined to the epithelium, transurethral resection should be curative.
- Primary malignant melanoma of the genitourinary tract is a rare lesion, accounting for only 0.2% of all melanomas. The urethra and the penis are the most frequent sites of origin. The bladder has been infrequently reported as a site of a primary melanoma [7, 38].
- Only 18 cases of primary melanoma of the urinary bladder were reported at the time of their publication in 2006 [4, 9].

Other authors have reported cases of melanoma of the urinary bladder as well as melanosis involving the urinary bladder [40–60]. Jin et al. [52] stated the following.

- Melanosis refers to abnormal or excessive deposition of melanin pigment in the cells and/or tissue, which can be seen in any organ but commonly in skin and oral mucosa.
- Melanosis of the urinary bladder is an extremely rare benign condition and only a handful of cases had been reported in the English literature before.
- Melanosis of the urinary bladder is a very rare benign condition and only 6 cases had been reported in the English literature prior to their reported case in 2009 [53–56].

Jin et al. [52] stated that they reviewed urothelial melanosis in the English literature and found the following. (a) All the reported patients had some associated symptoms with haematuria, voiding difficulty, or urinary incontinence. (b) The patients' ages ranged from 43 years to 86 years and both sexes were affected; (c) cystoscopically brown to black pigmentation of the urinary bladder mucosa with flat or punctate patterns was usually observed; (d) the pigmentation could be seen in any location in the bladder; (e) there had not been any description of associated mass lesions.

Rossen and Petersen [54] stated that in cases of melanosis (a) histologically, dark brown pigments were present mainly in the urothelial cells, but sometimes few pigment deposits were also seen in the lamina propria; (b) the pigments were nonrefractile and powdery and could form variable sized globules; (c) the pigments were positive by Fontana Masson stain and negative for PAS and Gomori's or Lillie's iron stains; (d) the melanin pigment disappeared after melanin bleach treatment; (e) in one case, melanocytes were seen admixed with urothelial cells appreciated by S100 protein immunohistochemical stain, but not in the Haematoxylin and Eosin sections; (f) they did not identify any nuclear atypia or melanoma in any of the described patients.

Tzankov et al. [57] iterated that (a) melanin pigments should be differentiated from lipofuscin and hemosiderin deposits; (b) lipofuscin deposits are referred to as lipofuscinosis or pseudomelanosis in order to separate them from true melanosis (melanin deposits). (c) lipofuscin forms brown fine granules which are usually perinuclearly located, representing undigested material from lipid peroxidation and they are associated with aging; (d) lipofuscin is positive by PAS stain negative by Fontana Mason and iron stains and does not disappear after bleaching procedure (bleaching resistant). Tzankov et al. [57] reported lipofuscinosis of the urinary bladder in a patient with interstitial cystitis after long-term treatment with ciprofloxacin. The urinary bladder showed scattered brownish discolorations at cystoscopy examination and the histological sections showed pigment granules in the urothelial cells and in the lamina propria histocytes. The pigment was PAS positive and bleach resistant. It was speculated that administration of ciprofloxacin might be the underlying cause of urinary lipofuscinosis due to free radicals generated by metabolism of ciprofloxacin. Furthermore, Berneis et al. [58] reported lipofuscinosis of the urinary bladder in patients with phenacetin abuse, which, interestingly, also promote generation of free radicals during metabolism.

Jin et al. [52] stated that, in comparison with lipofuscin deposit in the urinary bladder, lipofusion deposit is more commonly encountered in the colonic biopsy specimen as melanosis coli and are usually associated with anthracene containing laxative or herbal remedies usage. They also stated that the colonic mucosa usually shows brown discoloration under colonoscopy examination, and histologically, it shows pigmented macrophages in the lamina propria. Freeman [59] stated that the terminology of melanosis coli is misleading, because the pigments are not melanin at all. It has been suggested that, in this situation, the term pseudomelanosis coli or lipofuscinosis coli may be more appropriate to reflect the etiology of the colonic pigmentation [52].

Jin et al. [52] stated the following.

- Hemosiderin is an intracellular storage form of iron that is produced by phagocytic digestion of hematin.
- They appear as a golden yellow-brown intracellular or extracellular pigment under light microscopic examination, and pigments are positive by Gomori's or other iron stains.
- The excessive iron storage in tissue is known as hemosiderosis.

Engen and Herr [60] reported a case of hemosiderosis of urinary bladder in a man with multiple blood transfusions as part of treatment for his anaplastic anemia. Cystoscopy examination revealed a brown black 2.0 cm polypoid lesion on the right lateral wall and surrounding numerous smaller lesions of similar appearance. On Histological examination, the urothelial mucosa exhibited prominent hemosiderin deposits and hemosiderin-laden macrophages in the lamina propria with unremarkable urothelium.

Jin et al. [52] stated that, even though hemosiderin is mostly present in the stroma and macrophages, but not in the urothelial cells, it still should be included in the differential diagnosis for complete work-up [52].

Ainsworth et al. [7] stated that urinary melanosis needs to be differentiated from melanoma, which is also extremely rare in the urinary bladder and is classified as primary or metastatic, based on patient's clinical presentation and the presence or absence of atypical melanocytes in the urothelium. In the absence of cutaneous or other visceral melanoma and the presence of atypical melanocytes in the urothelium adjacent to melanoma, the tumour is classified as primary [52].

**Figure 1:**
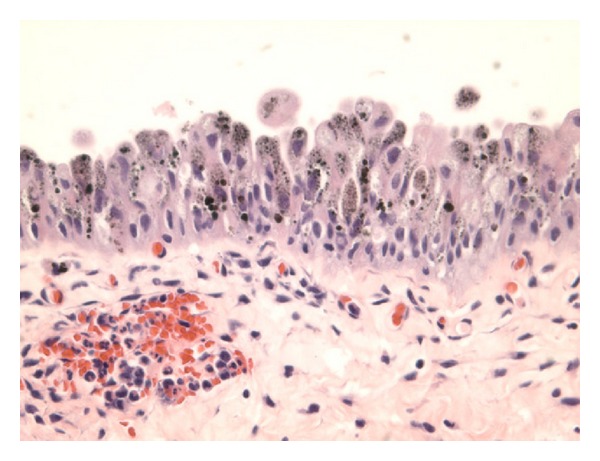
Haematoxylin and Eosin staining showing melanosis and not melanoma: this shows light brown powdery pigments to dark brown globules which are mainly present in the cytoplasm, partially or completely obscuring the nuclei. There is no evidence of any pigment deposit in the lamina propria. Taken from Jin et al. [52].

**Figure 2:**
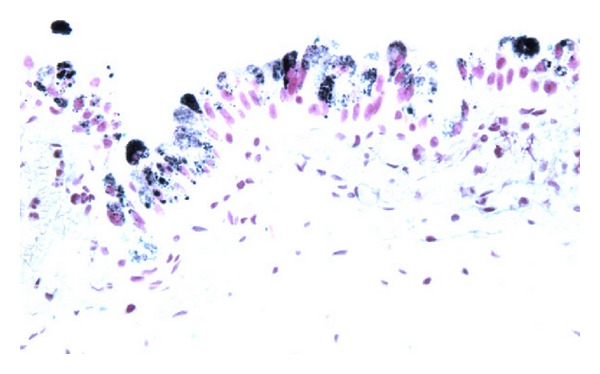
Fontana Masson stain for melanin: standard resolution. The pigments stain brown by Fontana stain which is consistent with melanin. Taken from Jin et al. [52].

Jin et al. [52] reported a 77-year-old woman with a history of urinary incontinence, large volume leakage, and severe urgency with leakage. She had been using diapers for the preceding two years. She was on Detrol for six months with no improvement in her symptoms. She underwent cystoscopy which revealed clusters of punctate, dark spots on the right lateral aspect of the floor of the urinary bladder. Biopsies were taken from the dark spots and her urine was sent for cytological examination. The pathology findings of the biopsy specimen consisted of tan soft tissues which ranged from 0.1 cm to 0.4 cm in greatest dimension. Microscopic examination of the sections revealed a benign urothelial mucosa with brown pigments focally present in the cytoplasm of urothelial cells. The pigments ranged from relatively light brown powdery to dark brown globules. The pigments were seen mainly in the cytoplasm and were partially or completely obscuring the nuclei. The underlying lamina exhibited congestion and acute inflammation, with no pigment deposit (see Figure 1). Special stains including Fontana Masson, Periodic Acid Schiff (PAS), and Gomori's Iron stain were performed. The pigments were found to be positive by Fontana stain which was consistent with melanin (see Figure 2), while the staining for PAS and Gomori's Iron was negative. The pigments disappeared after bleach procedure, further confirming the presence of melanin. There were no melanocytes identified in Haematoxylin and Eosin stained sections or immunohistochemical staining for S100 and HMB45 (human melanosome). The case was diagnosed as urinary melanosis. The urine cytology was negative for malignant cells and no pigmented urothelial cells were seen.

**Figure 3:**
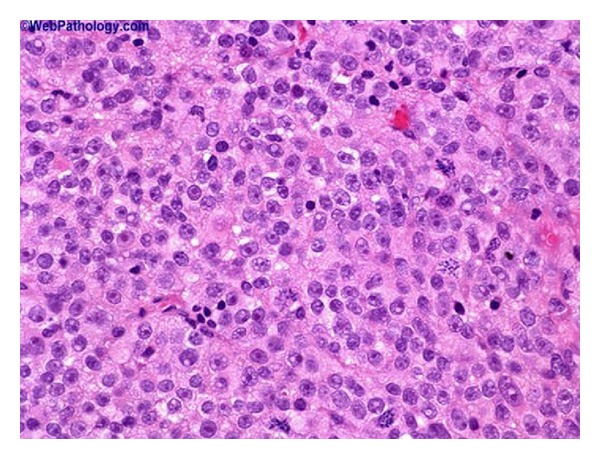
Bladder metastatic melanoma. Haematoxylin and Eosin stain: primary melanoma of the urinary bladder is very rare. In comparison, between 15% and 20% of patients who are dying of melanoma have urinary bladder involvement. This figure represents metastasis to the urinary bladder in a patient with known history of malignant melanoma, taken from Bladder Melanoma Pathology http://outlines.com/ with permission to reproduce the figure from info@biomedcentral.com.

**Figure 4:**
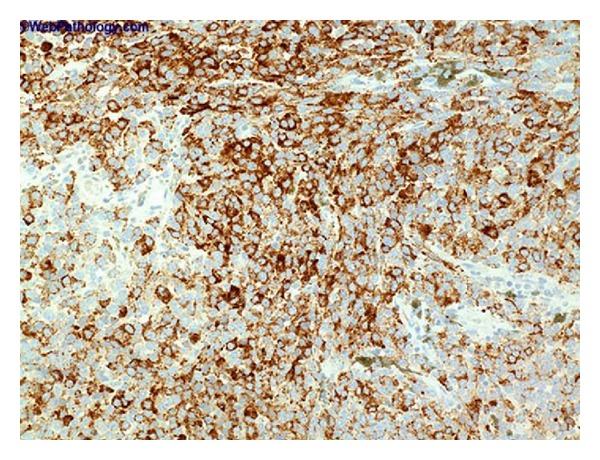
Bladder metastatic melanoma immunohistochemistry for HMB45. The differentiation of malignant melanoma from high-grade urothelial carcinoma may not be easy. Immunohistochemical staining for HMB-45 as depicted in the figure may help in the establishment of the correct diagnosis, taken from Bladder Melanoma Pathology http://outlines.com/, with permission to reproduce the figure from the original author source granted by info@biomedcentral.com.

De Torres et al. [44] reported a case report of primary melanoma of the urinary bladder in which the patient had gross hematuria for four months and black mass lesions of the urinary bladder were seen at cystoscopy examination. The histology examination of sections of the specimen showed spindle and epithelioid heavily pigmented malignant melanocytes with high mitotic rate (see Figure 3 for an example of the morphology of a melanoma of the urinary bladder which in this case was a metastatic melanoma taken elsewhere just for illustration purposes). Atypical melanocytes were also present in the urothelium. The tumour cells were positive for S100 protein and HMB45 (see Figure 4 for an example of positive staining with HMB-45). It was stated that being aware of clinical history, carefully reviewing histological features, and performing necessary immunohistochemical stains are very important in making the correct diagnosis. Urinary bladder melanosis is a benign condition of unknown clinical significance [44, 52].

Jin et al. [52] stated that one patient with melanosis of the urinary bladder was followed up for ten years and showed no development of malignancy [61]. Jin et al. [52] also stated that patients with melanosis, lipofuscinosis, hemosiderosis, or even melanoma of the urinary bladder can have similar nonspecific symptoms such as hematuria, cystitis, difficulty voiding, or urinary incontinence. They also stated that, at cystoscopy examination, all show pigmented lesions, although melanoma may have associated mass [52]. Jin et al. [52] iterated the following.

- Histological examination is the most important way to accurately classify the pigmented lesions in the urinary bladder.
- Melanosis of the skin or oral mucosa has been thoroughly studied because it is common. It can be seen in many conditions and is related to sun exposure (skin) or trauma (mucosa). It appears as gray or black macules. Histologically, it shows melanin deposits in the basal layer of the squamous epithelium.
- Melanosis of oral mucosa can also be seen in smokers and in patients with Peutz-Jeghers syndrome [62].

Jin et al. [52] stated that, unlike melanosis of the skin and oral mucosa, the underlying etiology of urinary melanosis is not yet established. Since no melanocytes are normally present in the urothelium or metaplastic epithelium, melanocyte ectopia has been speculated for the primary melanoma of urinary bladder [7] which may also apply to the development of urinary melanosis.

Some authors [7, 39, 63] stated that the theory of melanocyte embryology suggested how malignant melanoma might occur in a location as unusual as that of the urinary bladder. Ainsworth et al. [7] stated the following.

- Melanocytes migrate from their origin in the neural crest via the mesenchyme and ultimately deposit in skin and hair follicles.
- If these cells arrest in an ectopic site such as the developing urinary bladder, and subsequently go through neoplastic transformation in postnatal development, primary malignant melanoma of the urinary bladder would follow.

Niederberger and Lome [12] reported a 53-year-old man who presented with urinary frequency, urgency, and increasing nocturia. He was found to have Escherichia coli urinary tract infection which was treated with trimethoprim-sulfamethoxazole. Six months later he developed a recurrent infection and he underwent urological evaluation. The patient had a ten-year history of prostatitis which was treated with trimethoprim-sulfamethoxazole and prostatic massage. Eight years prior to his presentation a cutaneous pigmented lesion was excised from his left shoulder and histological examination was reported to exhibit a benign nevus. His urinalysis was normal. He had intravenous pyelogram which revealed a 5 cm lesion at the floor of the bladder and a thick-walled urinary bladder; the prostate and seminal vesicles were enlarged and irregular, and the periprostatic planes were obliterated which was adjudged to be suggestive of an infiltrative process; the pelvic lymph nodes were normal. He underwent cystoscopy which revealed a darkly pigmented lesion on the floor of the urinary bladder; the prostate gland was enlarged and the junction of the prostate with the urinary bladder appeared normal. He underwent trans-urethral resection of the pigmented urinary bladder lesion and 8 grams of tissue was resected. Histological examination of the resected lesion revealed pigmented malignant melanoma. He had extensive investigations to exclude other primary sites as the source of the melanoma. He underwent dermatologic and ophthalmologic evaluations which were negative. His serum biochemical tests and his serum enzymes were normal. He had upper gastrointestinal and barium enema studies which were normal. He also had computed tomography scan of abdomen, brain, and chest which were normal. A diagnosis of primary malignant melanoma of the urinary bladder was made. He underwent pelvic bilateral lymphadenectomy and frozen section examination of the specimen was negative for metastases. He then underwent a nerve-sparing radical cystectomy and prostatectomy and construction of an Indiana Pouch continent urinary diversion. By the time of publication of the case report by Niederberger and Lome [12], the patient was followed up for eighteen months without any evidence of locally recurrent or metastatic disease. Pathological examination of the gross specimen revealed

- a large bulky volume malignant melanoma which involved the urinary bladder and the prostate gland,
- darkly pigmented nodules, superior to the Denonvilliers fascia, on the posterior aspect of the urinary bladder which contained melanoma,
- the tumour was deeply invasive through the wall of the urinary bladder wall to, but not through, the peri-vesical fat,
- invasion of the tumour into the prostatic parenchyma,
- no evidence of tumour in the seminal vesicle,
- that the surgical margins were free of tumour,
- that sections of the obturator lymph node revealed micrometastases,
- that histological examination of the primary tumour was consistent with pigmented malignant melanoma.

Immunoperoxidase staining and electron microscopic examination of the specimen revealed intraepithelial melanocytes, separate from the pigmented tumour.

Niederberger and Lome [12] stated that the finding of intra-epithelial melanocytes in the urinary bladder mucosa separate from the pigmented urinary bladder tumour in their reported case would support the postulate that melanoma arose from ectopic melanocytes and that their findings also document the primary nature of this urinary bladder melanoma.

A number of treatment options have been used by a number of authors for the treatment of melanoma of the urinary bladder and these include trans-urethral resection, partial cystectomy, total cystectomy with urinary diversion, chemotherapy, and external beam radiotherapy singly or in combination [7–11]. Niederberger and Lome [12] stated that the following.

- Despite these disparate treatment modalities the prognosis had been dismal.
- In Ainsworth and associates' patient [7] who was treated by means of total cystectomy and subsequently by chemotherapy and radiotherapy, pelvic tumour recurrence developed at fourteen months.
- Their patient who was treated by radical cystectomy and prostatectomy with urinary diversion represented the longest tumour-free survivor, at eighteen months, without locally recurrent or metastatic disease.
- The poor prognosis of primary melanoma of the urinary bladder may be related to a delay in diagnosis. Deep muscle infiltration was reported in the pathology of all previously reported cases [7–11].
- The aggressive biopsy of any pigmented lesion found in the bladder may reveal nests of melanocytes which would identify patients in whom this rare malignant bladder tumour might develop. If such tumours could be treated before they became deeply infiltrative, the prognosis for primary melanoma of the urinary bladder might improve.

Sundersingh et al. [64] reported a 56-year-old man who complained of difficulty in voiding and haematuria of 1 month's duration. He was found elsewhere to have a growth in the bladder with an initial diagnosis of leiomyosarcoma. He underwent cystoscopy which revealed a friable, haemorrhagic tumour in the anterior wall of the urinary bladder. Histologic examination of his cystoscopic biopsy specimen showed transitional epithelium with an underlying tumour composed of fascicles and sheets of oval fusiform cells. The tumour cells had moderate cytoplasm and spindle shaped hyperchromatic nuclei with conspicuous nucleoli and more than 10 mitoses per 10 hpf. Immunohistochemical staining of the tumour showed positive reaction to the tumour cells for Vimentin, S100 protein, and Melan A and negative reaction for muscle actin, smooth muscle actin, desmin, and HMB-45. There was no history of regressed cutaneous melanoma and dermatological, gastrointestinal, and otorhinolaryngological examinations were negative. A diagnosis of primary amelanotic spindle cell melanoma of the urinary bladder was made. He underwent a radical cystectomy with orthotopic ileal neo-bladder construction. Macroscopic examination revealed a polypoidal necrotic, haemorrhagic tumour involving the anterior and lateral wall of the urinary bladder measuring 9 × 6 × 6 cm. Histopathological examination of the specimen confirmed the preoperative of spindle cell melanoma. The lining transitional epithelium was not involved and no junctional activity was evident. Four months later, he developed acute retention of urine. He underwent laparotomy which revealed pelvic recurrence; the resection of which showed histologic features similar to that of the primary tumour. He died six months later.

## 4. Conclusions

Primary malignant melanoma of the genitourinary tract is a rare lesion. The bladder has been infrequently reported as a site of a primary melanoma. Approximately less than 30 cases (in fact about 27 cases) have been reported to date. There is no consensus opinion regarding the treatment option that would achieve a good prognosis in the management of primary malignant melanoma of the urinary bladder perhaps because of the fact that very few cases have so far been reported. Almost all reported cases have been fatal; in view of this it would be recommended that oncologists and urologists should report their cases of primary melanoma of the urinary bladder and they should conduct a multicentre trial of treatment options aimed at improving the prognosis of this fatal lesion. Additionally urinary melanosis which is a differential diagnosis of melanoma is a benign lesion.

## Figures and Tables

**Table 1:** List of some of the cases of primary malignant melanoma of bladder reported in the literature with treatment and outcome (this list has 26 primary and one metastatic bladder melanoma; only one metastatic melanoma was included; there are more metastatic bladder melanomas but only 26 primary melanomas of the urinary bladder were found by the author).

Authors and publication references	Age (in years)	Sex	Treatment	Followup
Wheelock [8]	67	F	Partial cystectomy	Died, 36 months
Su and Prince [9]	61	F	None	Died, 2 months
Ainsworth et al. [7]	65	F	Cystectomy	Alive, 17 months
Willis et al. [11]	57	F	Radical cystectomy	Died, 36 months
Anichkov and Nikonov [10]	48	Male	Partial cystectomy	Died, 12 months
Anichkov and Nikonov [10]	46	Male	Cystectomy	Alive, 3 months
Ironside et al. [41]	56	Male	None	Died, 8 months
Goldschmidt et al. [46]	53	Female	Partial cystectomy	Died, 7 months
Goldschmidt et al. [46]	56	Female	None	Alive, 6 months
van Ahlen et al. [13]	81	Male	Cystectomy, Radiotherapy, Interferon-*α*	Died, 24 months
Lund et al. [49]	81	Female	Local excision, Radiotherapy-chemotherapy	Alive, 15 months
Kojima et al. [48]	63	Female	Chemotherapy	Died, 18 months
Lange-Welker et al. [43]	75	Male	Partial cystectomy	Died, 3months
Mourad et al. [51]	34	Male	Radical cystectomy	Alive, 12 months
De Torres et al. [44]	44	Male	Radical cystectomy	Died, 14 months
Tainio et al. [45]	52	Male	Trans urethral resection of lesion	Died, 8 months
García Montes et al. [40]	44	Female	Trans urethral resection	A, 144 months
Khalbuss et al. [4]	82	Female	Radiotherapy/cystectomy	Died, 16 months
Pacella et al. [5]	82	M	Transurethral resection of lesion	Died, 9 months
Charfi et al. [31]	54	Male	TURBT (for metastatic) Metastatic oesophageal melanoma not primary	Died, 1 month
Niederberger and Lome [12]	53	Male	TURBT, pelvic lymph Bilateral pelvic lymph adenectomy Radical cystectomy and Prostatectomy	Alive 18 months No recurrence
El Ammari et al. [6]	71	Male	TURBT (Transurethral resection) 5 cm sized mass	Died, 5 months
Philippe et al. [50]	77	Male	TURBT	Outcome not available to author
Baudet et al. [47]	7	Female	Partial cystectomy	Alive, 7 years
T. Hsu and Y. Hsu [42]	73	Male	TURBT and intravesical BCG TURBT for recurrence at 2, 7, and 9 months	Alive, 16 months
Sundersingh et al. [64]	56	Male	TURBT + radical cystectomy Resection of pelvic recurrence 4 months later	Died, 10 months
